# American Medical Association

**Published:** 1877-04

**Authors:** Wm. B. Atkinson

**Affiliations:** Permanent Secretary.; 1400 Pine Street, Philadelphia


AMERICAN MEDICAL ASSOCIATION.—The twenty-eighth annual session will be held in the city of Chicago, Ill., on Tuesday, June 5, 1877, in Farwell Hall, at 11 A.M.

"The delegates shall receive their appointment from permanently organized State medical societies, and such county and district medical societies as are recognized by *representation in their respective State societies*, and from the Medical Department of the Army and Navy of the United States."

"Each State, county, and district medical society entitled to representation shall have the privilege of sending to the Association one delegate for every ten of its regular resident members, and one for every additional fraction of more than half that number: *Provided*, however, that the number of delegates for any particular State, territory, county, city, or town shall not exceed the ratio of one in ten of the resident physicians who may have signed the Code of Ethics of the Association."

Secretaries of medical societies as above designated are earnestly requested to forward, *at once*, lists of their delegates, with residences, in order that a correct record may be made of all who are in affiliation with this body.

WM. B. ATKINSON, M.D.,
*Permanent Secretary.*

1400 Pine Street, Philadelphia.

